# Supplementary material for: Reduced Granule Cell Proliferation and Molecular Dysregulation in the Cerebellum of Lysosomal Acid Phosphatase 2 (ACP2) Mutant Mice
Source: Int J Mol Sci. 2021 Mar 15;22(6):2994. doi: 10.3390/ijms22062994 (PMC7999993; doi:10.3390/ijms22062994)
Supplement: Supplementary file 1 [file ijms-22-02994-s001.pdf]

# Supplementary Materials

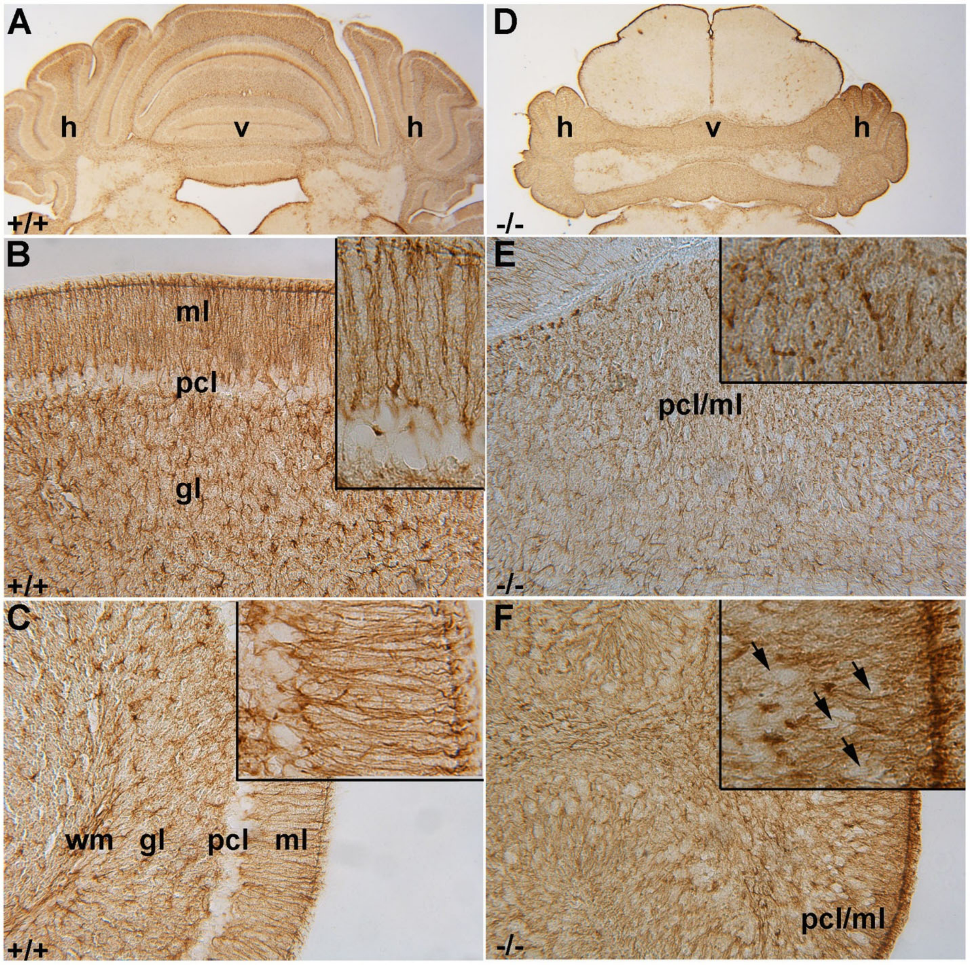


**Figure 1.** Bergmann glial cells are scattered in the *nax* cerebellum at P13. (**A**–**C**) Frontal sections of the wt cerebellum were immunostained with GFAP and showed astrocyte and Bergmann glial cell immunoreactivity. Bergmann glial cell bodies aligned with the PC layer and their fibers projected straight into the molecular layer in the vermis (**B**) and hemisphere (**C**) of the cerebellum in wt mice; (**B**,**C**) show the high magnification of (**B**,**C**). (**D**–**F**) Frontal sections of the *nax* cerebellum were immunostained for GFAP and showed astrocyte and Bergmann glial cell immunoreactivity. Bergmann glial cell bodies did not appear aligned in a straight line and fibers looked less organized, with some projecting to the pial surface in vermis (**E**). Bergmann glial cells appeared more organized in the hemisphere of the *nax* cerebellum (**F**) and located in between the ectopic Purkinje cells in the molecular layer (arrow). gl, granular layer; h, hemisphere; ml, molecular layer; pcl, Purkinje cell layer; v, vermis; wm, white matter. Scale bar = 500 μm in (**A**,**D**) and 100 μm in (**B**–**F**) and 50 μm in (**B**–**F**).

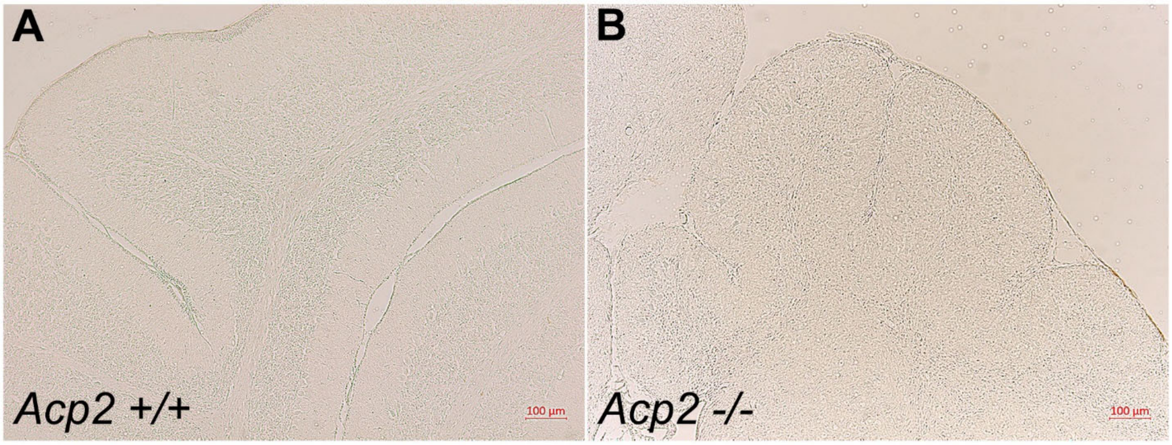


**Figure 2.** **A**,**B** Sagittal sections of P12 wt and *nax* mouse cerebella immunostained with secondary antibody. The secondary antibody control (no primary antibody control), run in parallel with SHH immunohistochemistry, shows that there is no nonspecific binding in wt (**A**) and *nax* (**B**) littermates.

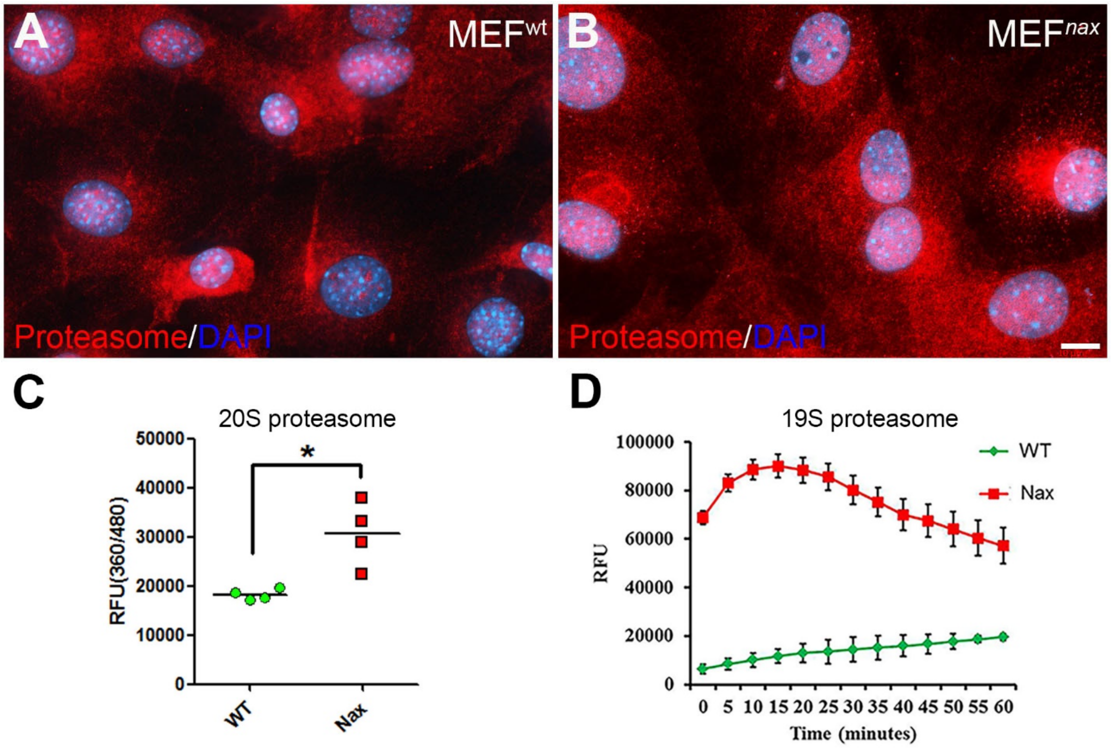


**Figure 3.** Proteasome expression and activities assessment in MEF$^{wt}$ and MEF$^{nax}$ cells. **A**,**B** MEF$^{wt}$ and MEF$^{nax}$ immunostained by anti-proteasome 20S alpha + beta (red) and DAPI (blue) show immunoreactivity in the nucleus and cytoplasm, with a similar expression pattern in the MEF$^{wt}$ and MEF$^{nax}$. **C**,**D** To measure 26S activity, the levels of both 19S (deubiquitinating activity) and 20S (proteolytic activity) were determined. The activity of both 20S (**C**) and 19S (**D**) subunits are significantly higher in MEF$^{nax}$ than in MEF$^{wt}$. The data in the graph are presented as the mean of three independent experiments (each experiment was repeated six times) ± SEM, and statistical analysis was performed using unpaired *t*-test ($p < 0.05$). Scale bars: 10 μm in **B** applies to **A**,**B**.
